# Leaf Water Storage and Robustness to Intermittent Drought: A Spatially Explicit Capacitive Model for Leaf Hydraulics

**DOI:** 10.3389/fpls.2021.725995

**Published:** 2021-10-14

**Authors:** Yongtian Luo, Che-Ling Ho, Brent R. Helliker, Eleni Katifori

**Affiliations:** ^1^Department of Physics and Astronomy, University of Pennsylvania, Philadelphia, PA, United States; ^2^Department of Biology, University of Pennsylvania, Philadelphia, PA, United States

**Keywords:** biophysical modeling, leaf hydraulics, vascular network, water stress, xylem, transpiration, water-storage capacitance, monocot

## Abstract

Leaf hydraulic networks play an important role not only in fluid transport but also in maintaining whole-plant water status through transient environmental changes in soil-based water supply or air humidity. Both water potential and hydraulic resistance vary spatially throughout the leaf transport network, consisting of xylem, stomata and water-storage cells, and portions of the leaf areas far from the leaf base can be disproportionately disadvantaged under water stress. Besides the suppression of transpiration and reduction of water loss caused by stomatal closure, the leaf capacitance of water storage, which can also vary locally, is thought to be crucial for the maintenance of leaf water status. In order to study the fluid dynamics in these networks, we develop a spatially explicit, capacitive model which is able to capture the local spatiotemporal changes of water potential and flow rate in monocotyledonous and dicotyledonous leaves. In electrical-circuit analogs described by Ohm's law, we implement linear capacitors imitating water storage, and we present both analytical calculations of a uniform one-dimensional model and numerical simulation methods for general spatially explicit network models, and their relation to conventional lumped-element models. Calculation and simulation results are shown for the uniform model, which mimics key properties of a monocotyledonous grass leaf. We illustrate water status of a well-watered leaf, and the lowering of water potential and transpiration rate caused by excised water source or reduced air humidity. We show that the time scales of these changes under water stress are hugely affected by leaf capacitance and resistances to capacitors, in addition to stomatal resistance. Through this modeling of a grass leaf, we confirm the presence of uneven water distribution over leaf area, and also discuss the importance of considering the spatial variation of leaf hydraulic traits in plant biology.

## 1. Introduction

Fluid flows in the plant vascular tissue system, which consists of xylem vessels for water transport and phloem vessels for the transport of photosynthetic products from leaves, are by no means isolated from other plant tissues. This is especially prominent in leaf hydraulic networks, which are typically the terminal portions of water flow through xylem. The xylem vessels making up these networks not only connect to phloem through leaf tissue, but also deliver water to an extra-xylary network of living cells from which water is evaporated and transpired to the atmosphere through leaf-surface pores (stomata) (Sack and Holbrook, 2006; Stroock et al., 2014). Photosynthetic carbon assimilation requires stomata to remain open for the exchange of carbon dioxide with air, while transpiration simultaneously leads to a large sum of water loss, resulting in water-use efficiency (CO_2_ uptake per water molecule loss) as low as 1/500 (Taiz and Zeiger, 2002). Implementation of water stress by a shortage of water source at the leaf base or decreasing atmospheric humidity around the leaf will cause stomata to close, thus suppressing transpirational water loss, but also reducing photosynthesis (Brodribb, 2009; Choat et al., 2018). The maintenance of leaf water status, which changes spatially in the xylem hydraulic vascular network, is therefore critical to keeping stomata open and sustaining photosynthesis.

Water storage functions of certain plant cells help to maintain plant water status, and supports the resilience and survival of a plant experiencing water stress (Tyree and Ewers, 1991; Jones, 2013). Succulent plants are perhaps the most obvious example, where water-storage parenchyma cells play the role of hydraulic capacitors, storing water when water supply is sufficient and providing water to sustain water status under stress (Smith et al., 1987). Most theoretical examinations of capacitance have focused on trees and how the substantial water storage in wood, ray parenchyma and embolized xylem conduits (Hölttä et al., 2009; Meinzer et al., 2009; Pfautsch et al., 2015; Mencuccini et al., 2017) offer a water source for continued transpiration and/or the maintenance of plant-water potential above safety margins during seasonal changes in soil or atmospheric drought (Salomón et al., 2017; Bryant et al., 2021). Less attention has been paid to the capacitance of leaves, and how local capacitance may buffer the transpiration stream through the relatively rapid vagaries of environment to which a leaf is subjected.

In grass leaves, bulliform cells, water-storage parenchyma, and vascular bundle sheaths could all play the role of dynamic local capacitors (Raven et al., 2005). Previous theoretical work on the water-storage capacitance was typically from the perspective of whole leaf or whole plant, such as in a lumped-element model using electrical-circuit analogs, where a whole system-wide capacitor is used as well as other whole-system elements including resistors (Jones, 1978). This whole-system approach extends also to the interpretation of pressure-volume curve measurements where capacitance, determined for both pre- and post-turgor-loss points, is necessarily assigned to the whole leaf and not specific cells (Bartlett et al., 2012). The more cell-specific, leaf-level models of fluid (and vapor) flow in leaves do not explicitly include water-storage capacitance (Buckley, 2015). Water reservoir cells are distributed along water pathways in the network, which means capacitance is spatially dependent and could affect the water status locally. Transpiration also occurs locally through stomata all over the leaf surface, making vessels in the network behave as leaking pipes. The competing effects of transpiration and water storage under stress will thus be more appropriately investigated in terms of spatially explicit network systems. In grass leaves, the unbalanced distribution of water content from leaf base to tip is illustrated by the fact that the area near tip is disproportionately disadvantaged and dries out faster than the area near the base (water source) when subject to water shortage or even a transient change in atmospheric humidity. Even in a static, fully hydrated environment, the uneven spatial water distribution in a grass leaf is measurable by gravimetric methods (see section 2), as illustrated in section 3.

The plant or leaf water status, generally described by water potential ψ which is regarded as the driving force of water flow, has been theoretically studied by two classes of models. In both classes, the water transport through leaf xylem is treated as laminar following the Hagen-Poiseuille law, in which the hydraulic resistance of a xylem vessel is equal to the water potential difference between its two ends divided by the flow rate (van den Honert, 1948; Altus et al., 1985; Tyree and Ewers, 1991). The first type of model considers the small-scale spatial variations of leaf vascular networks, by implementing a network system consisting of only resistors, while ignoring the water-storage capacitance, for both monocot (Wei et al., 1999; Martre et al., 2001) and dicot (Cochard et al., 2004; Katifori, 2018) leaf modeling. In the second modeling type, large-scale tissue or organ-level properties including both resistance and capacitance are considered, while ignoring any spatially explicit architecture within a leaf (Cowan, 1965, 1972; Smith et al., 1987; Steppe et al., 2006). In such a model, the water flow through leaf or plant is commonly driven by a current source representing transpiration, which can be adjusted arbitrarily or according to experimentally measured transpiration rate, without an explicit, direct mechanistic input from water potential deficit (or vapor pressure deficit) between the leaf and air. Here, we bridge these two classes of models by developing a spatially explicit leaf hydraulic network model with local capacitance. While our model is general and can be applied to any type of vascularized leaf, we focus on grass leaf examples as they almost ubiquitously have water storage cells, and the parallel vein structure of grasses leads to water being lost throughout the length of the blade. While this too occurs in dicots over short distances (Zwieniecki et al., 2002), the process occurs along the entirety of a grass leaf. By conducting computation and simulation on a uniform grass leaf model, we study the dependence of transpiration rate on leaf hydraulic traits and water potentials in the environment.

We illustrate and discuss how capacitance increases the robustness of a leaf in a changing environment and maintains leaf water status, so that stomata can remain open to potentially sustain photosynthesis along the entire leaf blade. To examine this, we assume a condition that might appear unorthodox to a plant physiologist, that of static stomatal resistance. While constant stomatal resistance is not typically observed, this is not simply an academic assumption or a model convenience. First, rapid changes in leaf-to-air evaporative gradients, such as those coming from increases in wind speed, can expose the leaf to high transpirational demands before stomata respond by closing. Second, not all stomata respond the right way. A large number of species display a “wrong-way” stomatal response and temporarily increase opening with increasing evaporative demand, a response that can last 10s of minutes and further exacerbate transpirational demand (Buckley et al., 2011). In such scenarios, local capacitance could conceivably allow for continued transpiration and photosynthesis without dropping leaf-water potentials to damaging levels. Our modeling results, which are based on idealized theoretical assumptions, are not directly comparable with any known experimental measurements of spatial variations of water potential, but can be indirectly validated by our gravimetric measurements of water content distribution in the leaves of grass *Anthaenantia villosa*. See section 4 for discussions on experimental validation and biological applications.

## 2. Methods of Theoretical Modeling and Experimental Measurement

### 2.1. The Spatially Explicit Model of Capacitive Leaf Hydraulics

**Figure 1:**
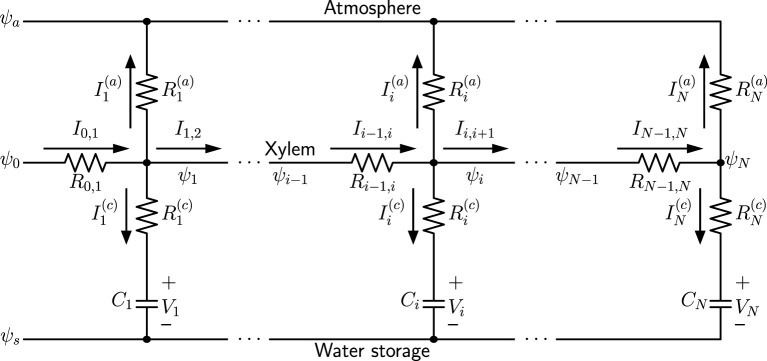
A one-dimensional capacitive network model, where ψ_*a*_ is the water potential in the atmosphere, ψ_*s*_ is the baseline osmotic potential of water storage, ψ_0_ is the water source potential at the leaf base, and ψ_*i*_ (*i* = 1, 2, …, *N*) are water potentials at different nodes of the xylem. Currents $I_{i}^{\left(a\right)}$ are transpiration currents through stomata, and $I_{i}^{\left(c\right)}$ are charging capacitors *C*_*i*_, which represent leaf water storage function.

We use a capacitive electrical circuit analog to model the spatial variation of hydraulic traits of a simple plant leaf model (such as a monocot leaf). A one-dimensional network model analogous to an electrical circuit is illustrated in Figure 1, consisting of nodes *i* = 1, 2, …, *N*. In this example only one xylem conduit is shown as the midline. The water potential in the atmosphere ψ_*a*_, which is more negative than the water potentials in the xylem (ψ_0_, ψ_1_, …, ψ_*N*_), is related to relative humidity (RH) in the air through the relationship:

(1) $$\begin{array}{r} \psi_{a}=\frac{\bar{R}T}{v}\ln\left(\frac{\text{RH}}{100\%}\right) \end{array}$$

with ideal gas constant $\bar{R}=$8.3145 J·mol^−1^·K^−1^, room temperature *T* =298.15 K, and liquid water molar volume *v* ≈ 18 mL/mol (Buckley and Sack, 2019). The water potential ψ_*s*_ underneath capacitors is the baseline osmotic potential of leaf water storage, which is specifically defined as the plant root potential plus the most negative osmotic potential of water-storage cells, when the cell water content is at the minimum for the cells to behave like linear capacitors. With this definition, ψ_*s*_ is always more negative than water potential ψ_*i*_ in the xylem, and the voltage *V*_*i*_ across each capacitor *C*_*i*_ is considered to be the positive hydrostatic pressure (turgor) (Smith et al., 1987; Jones, 2013), for which the plus and minus signs label the direction of *V*_*i*_ in the diagram. We assume the atmospheric condition outside the leaf and the baseline osmotic potential of reservoir cells inside are both uniform along the leaf blade, and thus have a single wire for ψ_*a*_ and ψ_*s*_, respectively in the diagram.

Figure 1 represents a well-watered leaf, where the water source potential ψ_0_ at the leaf base keeps charging the water-storage capacitors *C*_*i*_, and stomata are wide-open so that water is being released into the atmosphere through transpiration. When the direction of current $I_{i}^{\left(c\right)}$ is reversed, the corresponding capacitor is discharged and loses water content, while the polarity of its voltage *V*_*i*_ does not change. The resistors $R_{i}^{\left(c\right)}$ represent the resistance in the water-storage pathways, and resistors $R_{i}^{\left(a\right)}$ are the total resistance from xylem to the atmosphere, including outside-xylem resistance (mainly through mesophyll) for liquid water inside the leaf and stomatal resistance for water vapor. At steady state, the capacitors are not being charged and all $I_{i}^{\left(c\right)}=0$, when the water status is in equilibrium and turgor and water content are at the maximum. When stomata are closed, $R_{i}^{\left(a\right)}\to \infty$ and all $I_{i}^{\left(a\right)}=0$. The summation of all $I_{i}^{\left(a\right)}$ gives the total transpiration current $E=\underset{i}{\sum }I_{i}^{\left(a\right)}$.

The fundamental equations of the electrical analog are:

(2) $$\begin{array}{r} I_{i-1,i}=I_{i,i+1}+I_{i}^{\left(a\right)}+I_{i}^{\left(c\right)} \end{array}$$

(3) $$\begin{array}{r} \psi_{i-1}-\psi_{i}=R_{i-1,i}I_{i-1,i} \end{array}$$

(4) $$\begin{array}{r} \psi_{i}-\psi_{a}=R_{i}^{\left(a\right)}I_{i}^{\left(a\right)} \end{array}$$

(5) $$\begin{array}{r} I_{i}^{\left(c\right)}=\frac{\partial }{\partial t}\left[C_{i}\left(\psi_{i}-\psi_{s}-R_{i}^{\left(c\right)}I_{i}^{\left(c\right)}\right)\right]. \end{array}$$

At the terminal note (end of the xylem), $I_{N-1,N}=I_{N}^{\left(a\right)}+I_{N}^{\left(c\right)}$. With time-independent *C*_*i*_ and $R_{i}^{\left(c\right)}$, the last equation becomes:

(6) $$\begin{array}{r} I_{i}^{\left(c\right)}=C_{i}\frac{\partial \psi_{i}}{\partial t}-C_{i}R_{i}^{\left(c\right)}\frac{\partial I_{i}^{\left(c\right)}}{\partial t}. \end{array}$$

If we also assume the transpiration resistance $R_{i}^{\left(a\right)}$ is time-independent, we can obtain the following equation by substituting Expressions (4) and (6) into the first derivative of Equation (2) with respect to time ($\partial I_{i-1,i}/\partial t=\partial I_{i,i+1}/\partial t+\partial I_{i}^{\left(a\right)}/\partial t+\partial I_{i}^{\left(c\right)}/\partial t$):

(7) $$\begin{array}{r} \frac{\partial I_{i-1,i}}{\partial t}-\frac{\partial I_{i,i+1}}{\partial t}=\left(\frac{1}{R_{i}^{\left(a\right)}}+\frac{1}{R_{i}^{\left(c\right)}}\right)\frac{\partial \psi_{i}}{\partial t}\\ -\frac{1}{C_{i}R_{i}^{\left(c\right)}}\left(I_{i-1,i}-I_{i,i+1}-\frac{\psi_{i}-\psi_{a}}{R_{i}^{\left(a\right)}}\right). \end{array}$$

We will demonstrate how such a spatially uniform, one-dimensional network can be treated as a continuous model when the number of nodes *N* is large, and can be studied through analytical calculation under certain circumstances. We will also design a numerical method to simulate both the steady state and the time-dependent behavior of a general capacitive network model, which is not necessarily uniform or one-dimensional.

### 2.2. Analytical Calculation of the One-Dimensional Model

We assume the size of a node *l* is small compared to the total length of the network *L*, so that the number of nodes *N* = *L*/*l* is large. At node *i* we define a normalized location *x* = *i*/*N* (Δ*x* = *l*/*L* so that 0 ⩽ *x* ⩽ 1) which changes continuously, and in the xylem the water potential ψ and current *I* also change continuously which means (ψ_*i*−1_ − ψ_*i*_)/Δ*x* → −∂ψ/∂*x* and (*I*_*i*−1,*i*_ − *I*_*i,i*+1_)/Δ*x* → −∂*I*/∂*x*. In this normalized continuous model, we assume the resistances and capacitances are uniformly distributed and time-independent throughout the network ($R_{i-1,i}=R^{\left(o\right)}$, $R_{i}^{\left(a\right)}=R^{\left(a\right)}$, $R_{i}^{\left(c\right)}=R^{\left(c\right)}$ and $C_{i}=C^{\left(o\right)}$ are all constants), and we define *R* = *NR*^(*o*)^, $R_{a}=R^{\left(a\right)}/N$, $R_{c}=R^{\left(c\right)}/N$ and *C* = *NC*^(*o*)^ as combined parameters for the whole system. Considering ∂ψ/∂*x* = −*RI*, we derive the following basic equation from Equation (7):

(8) $$\begin{array}{r} \frac{\partial^{3}\psi }{\partial t\partial x^{2}}=R\left(\frac{1}{R_{a}}+\frac{1}{R_{c}}\right)\frac{\partial \psi }{\partial t}-\frac{1}{CR_{c}}\frac{\partial^{2}\psi }{\partial x^{2}}+\frac{R}{CR_{c}R_{a}}\psi -\frac{R\psi_{a}}{CR_{c}R_{a}}. \end{array}$$

We outline the general time-dependent solution of this equation in Supplementary Section 1. The steady-state average potential in the xylem is:

(9) $$\begin{array}{r} \bar{\psi }=I_{0}R_{a}+\psi_{a} \end{array}$$

where *I*_0_ = *I*(*x* = 0) is the current entering through the base (see Equation S8 in Supplementary Material for expression), which is equal to the total transpiration current *E* at steady state.

For a nontrivial, time-dependent water potential boundary condition (dynamic ψ_0_ or ψ_*a*_ induced by the change of water supply or air humidity), in Supplementary Section 2 we illustrate the solution for a dynamic water source ψ(*x* = 0, *t*) = *A* cos(ω_0_*t* + φ) that oscillates with time. We also consider an excised leaf xylem which is initially at the fully hydrated steady state and is cut off at the leaf base at *t* = 0, when the base water source is turned off. The calculation is detailed in Supplementary Section 3. It turns out that both average xylem potential $\bar{\psi }$ and total transpiration current *E* are exponential decay functions with time at *t* > 0, where time constant is τ = *C*(*R*_*c*_ + *R*_*a*_):

(10) $$\begin{array}{r} \bar{\psi }\left(t\right)=\psi_{a}+I_{0}R_{a}\exp\left(-\frac{t}{C\left(R_{c}+R_{a}\right)}\right) \end{array}$$

(11) $$\begin{array}{r} E\left(t\right)=I_{0}\exp\left(-\frac{t}{C\left(R_{c}+R_{a}\right)}\right) \end{array}$$

which means the existence of capacitance *C* as well as transpiration resistance *R*_*a*_ (which is static) and water-storage pathway resistance *R*_*c*_ slows down the process of losing water in a drought condition where the leaf loses its water source, illustrating the function of capacitance in a plant's resilience against drought.

**Figure 2:**
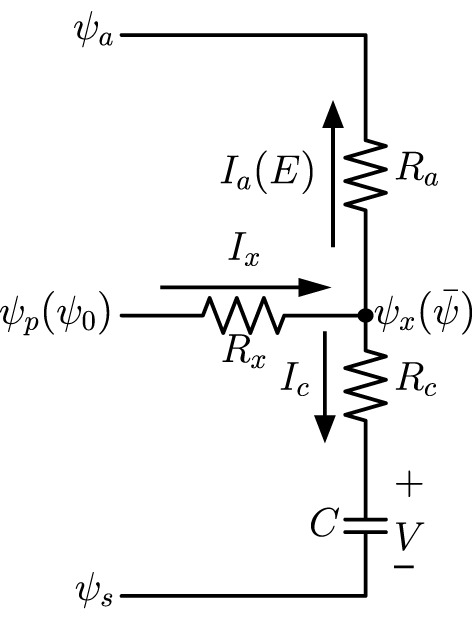
A lumped-element model which is equivalent to the one-dimensional network in Figure 1. Here ψ_*p*_ = ψ_0_ is the water source potential at the leaf base, and $\psi_{x}=\bar{\psi }$ is the average potential in the xylem. Currents *I*_*a*_ = *E* and *I*_*c*_ are total transpiration current and capacitor charging current, respectively.

A highly lumped-element model can be derived from the one-dimensional network in Figure 1 and validated by analytical calculations of the continuous model. The diagram of the model, which is similar to lumped models commonly found in literature on whole-plant or whole-leaf modeling, is in Figure 2, in which the water source potential ψ_0_ at the leaf base is relabeled as ψ_*p*_, ψ_*x*_ is the average water potential in the xylem ($\bar{\psi }=\underset{i}{\sum }\psi_{i}/N$), and *V* is the average capacitor voltage ($\bar{V}=\underset{i}{\sum }V_{i}/N$). The current *I*_*x*_ is the incoming current *I*_0_ through leaf base, while $I_{a}=\underset{i}{\sum }I_{i}^{\left(a\right)}$ is the total transpiration current *E* and $I_{c}=\underset{i}{\sum }I_{i}^{\left(c\right)}$ is the total capacitor charging (or discharging) current. With *R*_*a*_ and *R*_*c*_ defined previously as grouped elements for the whole system, we demonstrate in Supplementary Section 4 the equivalence of the lumped model in Figure 2 and the uniform model based on Figure 1, for which we need to define the effective xylem hydraulic resistance *R*_*x*_ = *R*/3 where *R* is the total xylem resistance. We also show that the time constant τ = *C*(*R*_*c*_ + *R*_*a*_) of an excised leaf can be obtained from the lumped model. When studying changing stomata whose conductance depends on leaf water status, we can use this lumped model to investigate the importance of stomatal sensitivity to local water content in transpiration control. See section 4 for relevant discussions.

### 2.3. Numerical Simulation of General Capacitive Networks

The one-dimensional model in Figure 1 can be generalized into two- or higher-dimensional networks, which can include branches and loops. If node *i* is a node in such a network, and its neighboring nodes are labeled by *n*(*i*), we use *I*_*n*(*i*),*i*_ and *R*_*n*(*i*),*i*_ to label the current from *n*(*i*) to *i* and the resistance between *n*(*i*) and *i*, respectively. Starting from the current relation:

(12) $$\begin{array}{r} \underset{n\left(i\right)}{\sum }\frac{\partial I_{n\left(i\right),i}}{\partial t}=\frac{\partial I_{i}^{\left(a\right)}}{\partial t}+\frac{\partial I_{i}^{\left(c\right)}}{\partial t}, \end{array}$$

we derive the water potential relation at *i*:

(13) $$\begin{array}{r} \left(\underset{n\left(i\right)}{\sum }\frac{1}{R_{n\left(i\right),i}}+\frac{1}{R_{i}^{\left(a\right)}}+\frac{1}{R_{i}^{\left(c\right)}}\right)\frac{\partial \psi_{i}}{\partial t}-\underset{n\left(i\right)}{\sum }\frac{1}{R_{n\left(i\right),i}}\frac{\partial \psi_{n\left(i\right)}}{\partial t}\\ \;=\frac{1}{C_{i}R_{i}^{\left(c\right)}}\left(\frac{\psi_{a}-\psi_{i}}{R_{i}^{\left(a\right)}}+\underset{n\left(i\right)}{\sum }\frac{\psi_{n\left(i\right)}-\psi_{i}}{R_{n\left(i\right),i}}\right) \end{array}$$

where ψ_*n*(*i*)_ is the water potential at node *n*(*i*). The problem of solving the time-dependent behavior of ψ_*i*_ is organized into a matrix equation ***Ax*** = ***b***, where at time *t*, the vector to be solved is:

(14) $$\begin{array}{r} x={\left(\frac{\partial \psi_{1}}{\partial t},\frac{\partial \psi_{2}}{\partial t},\ldots ,\frac{\partial \psi_{i}}{\partial t},\ldots ,\frac{\partial \psi_{N}}{\partial t}\right)}^{\text{T}} \end{array}$$

which contains the time derivatives of water potential at all nodes. The *i*th element of vector ***b*** is:

(15) $$\begin{array}{r} b_{i}=\frac{1}{C_{i}R_{i}^{\left(c\right)}}\left(\frac{\psi_{a}-\psi_{i}\left(t\right)}{R_{i}^{\left(a\right)}}+\underset{n\left(i\right)}{\sum }\frac{\psi_{n\left(i\right)}\left(t\right)-\psi_{i}\left(t\right)}{R_{n\left(i\right),i}}\right) \end{array}$$

and the elements in the invertible and symmetric matrix ***A*** are determined by:

(16) $$\begin{array}{l} A_{i,j}=\{\begin{array}{ll} \sum_{n\left(i\right)}1/R_{n\left(i\right),i}+1/R_{i}^{\left(a\right)}+1/R_{i}^{\left(c\right)} & i=j\\ -1/R_{j,i} & j\text{ is neighbor of }i\\ 0 & i\neq j\&j\text{ is not neighbor of }i \end{array}. \end{array}$$

If the node *i* is connected to one or more water sources, the external water potentials must be included in the evaluation of ***b***_*i*_ and ***A***_*i, j*_. For example, if node *i* is connected to a base potential ψ_*p*_, we have $b_{i}=1/\left(C_{i}R_{i}^{\left(c\right)}\right)\left[\left(\psi_{p}-\psi_{i}\right)/R_{p,i}+\left(\psi_{a}-\psi_{i}\right)/R_{i}^{\left(a\right)}+\underset{n\left(i\right)}{\sum }\left(\psi_{n\left(i\right)}-\psi_{i}\right)/R_{n\left(i\right),i}\right]$ and $A_{i,i}=1/R_{p,i}+\underset{n\left(i\right)}{\sum }1/R_{n\left(i\right),i}+1/R_{i}^{\left(a\right)}+1/R_{i}^{\left(c\right)}$, where *R*_*p,i*_ is the resistance between *i* and base (location of water source).

To simulate the dynamics of the network, we start from an initial water status ψ_*i*_(*t* = 0), calculate ***b*** and then ***x*** = ***A***^−1^***b*** at the current time *t*, and update the water potentials after a small time step Δ*t*:

(17) $$\begin{array}{r} \psi_{i}\left(t+\Delta t\right)=\psi_{i}\left(t\right)+\frac{\partial \psi_{i}}{\partial t}\Delta t. \end{array}$$

To numerically calculate the steady state where all ∂ψ_*i*_/∂*t* = 0, we organize the equation $\left(\psi_{a}-\psi_{i}\right)/R_{i}^{\left(a\right)}+\underset{n\left(i\right)}{\sum }\left(\psi_{n\left(i\right)}-\psi_{i}\right)/R_{n\left(i\right),i}=0$ into another matrix equation ***By*** = ***a***, where ***y***_*i*_ = ψ_*i*_, $a_{i}=\psi_{a}/R_{i}^{\left(a\right)}$ (or $a_{i}=\psi_{p}/R_{p,i}+\psi_{a}/R_{i}^{\left(a\right)}$ if *i* is connected to a water source ψ_*p*_), and ***B***_*i,j*_ = ***A***_*i,j*_ if *i* ≠ *j* while $B_{i,i}=\underset{n\left(i\right)}{\sum }1/R_{n\left(i\right),i}+1/R_{i}^{\left(a\right)}$ (or $B_{i,i}=1/R_{p,i}+\underset{n\left(i\right)}{\sum }1/R_{n\left(i\right),i}+1/R_{i}^{\left(a\right)}$). By solving ***y*** = ***B***^−1^***a*** we can calculate the steady-state water potentials in the network.

### 2.4. Experimental Measurements of the Spatial Distribution of Water Content Along Grass Leaf Blades

As an indirect validation of our theoretical modeling prediction that water content decreases spatially from leaf base to tip (because of the tapering of water potential), the C4 grass species *Anthaenantia villosa* (*A. villosa*) was used for measuring the local distribution of water amount in its leaves. For light treatment, the species was exposed to light (~500 μmol·m^−2^·s^−1^) for 1 h prior to the measurement. For dark treatment, the species was kept in the lab without a light source. Leaf lengths were measured and divided into five segments of equal length marked with sharpies. Sample leaves were cut sequentially from the tip to the base and placed in bags with moist air (except for the dark + 1 h equilibrium treatment). Leaf fresh mass (FM, g) was measured soon after leaf excision. Segments were dried in a 40 °C oven for >24 h for leaf dry mass (DM, g) measurement. For the dark + 1 h equilibrium treatment, the whole leaf was cut at the base and placed in double bags with moist air for 1 h in the dark prior to the procedure described above. The purpose of the dark + 1 h equilibrium treatment was to compare with the dark treatment results to rule out any possible statistical difference caused by extra equilibration after excision. (The leaves equilibrated for an extra hour after excision do not show statistically significant difference from dark treatment results in Supplementary Figure 7). The measurement result of water amount per unit dry mass (= (FM−DM)/DM, g/g) is calculated for each segment, used to characterize the spatial variations of water content along leaf blades to account for the effects of both leaf area and thickness on water storage.

## 3. Computation and Simulation Results of Uniform Model Leaf

### 3.1. The Well-Watered Steady State of a Uniform Leaf

**Figure 3:**
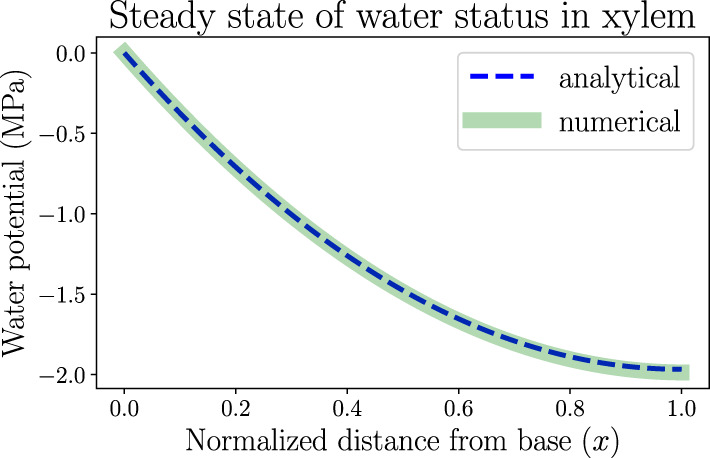
The spatially dependent water potential in a living, uniform model leaf at the steady state. The water potential decreases from 0 (*x* = 0, the leaf base) to about −2MPa (*x* = 1, the leaf tip). The one-dimensional numerical simulation result follows the analytical expression. Parameters used in the modeling are listed in Table 1. The steady-state transpiration rate of this model leaf is *E* = 1.97 mmol · *m*^−2^ · *s*^−1^.

In this section we apply the analytical calculation (for one-dimensional continuous models) and numerical simulation methods (for discretized network models) introduced in section 2 to the study of hydraulic behaviors of a model leaf, by making use of biologically relevant parameters. One of these results is the spatially dependent water potential profile at the steady state of a living uniform leaf shown in Figure 3. We assume the plant is well watered and estimate that the water potential at the leaf base ψ(*x* = 0) is approximately ψ_0_ = 0 where *x* is the normalized distance from base (*x* = 0) to tip (*x* = 1). In order to generate a clear spatial pattern of water status with significant spatial variations, we select an atmospheric water potential ψ_*a*_ =−100 MPa, which represents moderately dry air conditions outside the leaf stomata. Through Equation (1), the relative humidity is estimated to be RH ≈ 48%. The RH can be used to calculate the vapor pressure deficit (VPD) between the inner air space of the leaf and the outside atmosphere (across stomata), which is estimated to be VPD = (1 − RH)*P* ≈ 1.64 kPa, where *P* =3.17 kPa is the saturation vapor pressure of water (RH = 100%) at room temperature. The concept of VPD is usually used in plant biology as the driving force of transpiration.

Other modeling parameters used in this section, though not directly found in previous measurements, are selected to mimic the general trends of static or dynamic behaviors of leaf hydraulics. For example, the resulting steady-state water potential profile in Figure 3 illustrates a monotonically decreasing trend from base to tip, so that the water flow is unidirectional in this uniform model leaf. With the hydraulic resistance parameters we choose, including xylem total resistance *R* =2 MPa·m^2^·s·mmol^−1^ and resistance from xylem to air *R*_*a*_ =50 MPa·m^2^·s·mmol^−1^ (so that xylem conductance is 25 times the conductance into air), the leaf water potential is maintained above −2 MPa, below which xylem conduits may embolize and flow may start to cease. The analytical solution in Figure 3 as obtained from Equation S3 in Supplementary Material is a monotonically decreasing function. The numerical simulation of network is conducted by discretizing the leaf into *N* = 100 nodes from base to tip. The parameters for each node are derived from whole system parameters through the relationships introduced in subsection 2.2, e.g., *R*^(*o*)^ = *R*/*N* and $R^{\left(a\right)}=NR_{a}$. The simulation result reproduces the spatial distribution of water potential described by the analytical solution, also validating the usefulness of the simulation method which can be used for a more general, expanded network model. With these parameters, the average xylem water potential and total transpiration rate are calculated as $\bar{\psi }\;=$−1.31 MPa and *E* = *I*_0_ =1.97 mmol·m^−2^·s^−1^ according to Equations S7 and S8 in Supplementary Material, which are of the same order of magnitude as typical experimental values (which can be found in textbooks like Jones, 2013). If the distribution of water-storage capacitance is also uniform along the leaf, the local leaf water content will also similarly taper toward the tip, which can be tested by our gravimetric measurements. Parameters used in all the modeling work in this section are summarized in Table 1.

**Table 1:** Summary of modeling parameters used in this section.

**Parameter**	**Steady state (3.1)**	**Excised leaf (3.2)**	**Changing VPD (3.3)**
Water source potential ψ_0_ (MPa)	0	Removed	0
Atmospheric potential ψ_*a*_ (MPa)	−100	−100	−50 or −150
Baseline osmotic potential ψ_*s*_	Not used	Not used	Not used
Xylem hydraulic resistance *R* (MPa· m^2^· s· mmol^−1^)	2	2	2
Xylem-to-air resistance *R*_*a*_ (MPa· m^2^· s· mmol^−1^)	50	50	50
Water-storage capacitance *C* (mmol· m^−2^· MPa^−1^)	Not used	30 or 120	60 or 120
Xylem-to-capacitor resistance *R*_*c*_ (MPa· m^2^·s· mmol^−1^)	Not used	1 or 50	25 or 50

### 3.2. Exactly Solvable Model: Dehydration of an Excised Leaf

**Figure 4:**
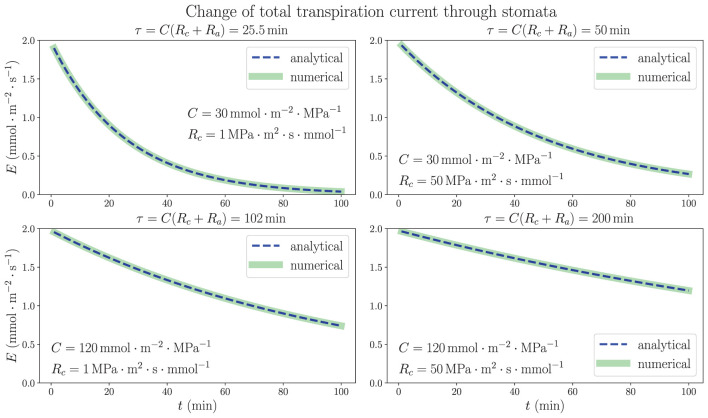
The time dependence of total transpiration rate through stomata when a living, uniform model leaf in steady state is removed from a plant and the water source is closed at the base. The 1D simulation results match the analytical exponential decay expressions, with time constants τ = *C*(*R*_*c*_ + *R*_*a*_). The temporal variations of average water potential in xylem are similar. Various sets of whole-leaf capacitance *C* and xylem-to-capacitor resistance *R*_*c*_ values are used, while other parameters are the same as those in Figure 3. The effect of increasing (or decreasing) the capacitance *C* on the rate of change is shown to be much larger than that of the increase (or decrease) of *R*_*c*_.

We consider the analytically solved situation of excising a living uniform model leaf in steady state from plant (see Supplementary Section 3). The results in Equations (10) and (11) show that the time dependence of both average xylem water potential and total transpiration current is characterized by a time constant τ = *C*(*R*_*c*_ + *R*_*a*_) in an exponentially decaying trend in the dehydration process, as long as the stomatal resistance is kept unchanged. Figure 4 illustrates the time dependence of total transpiration rate *E*, which continuously decreases from the steady-state value 1.97 mmol m^−2^ s^−1^ instead of going through a drastic change because of the existence of capacitance. In addition to the same parameters ψ_*a*_, *R* and *R*_*a*_ used in Figure 3, we use different sets of capacitance value *C* and resistance *R*_*c*_ from xylem to capacitor for the whole leaf as labeled in each subplot of Figure 4. The discretized numerical results, which are obtained by conducting the simulation in a 100-node network where *C*^(*o*)^ = *C*/*N* and $R^{\left(c\right)}=NR_{c}$ at each node (relationships from subsection 2.2 where *N* = 100) with time interval Δ*t* = 0.01 *min*, match the analytical expressions with time constant τ, revalidating the simulation method. These results are based on the critical assumption that all hydraulic elements are constant including transpiration resistance *R*_*a*_, which leads to a large drop of average xylem water potential $\bar{\psi }$ to very negative, non-physiological values in a matter of minutes (see Supplementary Figure 1). The decrease of transpiration rate, which would become nearly 0 ultimately as $\bar{\psi }$ drops to nearly ψ_*a*_, is entirely induced by the huge decline of $\bar{\psi }$ instead of closing stomata, as the *E*–$\bar{\psi }$ plot shows in Supplementary Figure 2. The calculations shown here are mainly used to explore the function of capacitors in the adjustment of leaf water status, emphasizing their importance for the stabilization and resilience of plant hydraulics, while ignoring other factors such as *R*_*a*_.

As predicted by the theoretical calculations and proven by the numerical simulations, both capacitance *C* and resistance *R*_*c*_ are shown to play an important role in the dehydration dynamics of a model leaf experiencing the removal of water source, which models the behavior of plant water status in a severe drought condition. It turns out that within the ranges of parameters we choose, the variation of *C*, which is directly proportional to τ, would exert a much larger influence on the time scale and rate of change in the dehydration process than the effect of varying *R*_*c*_. While *R*_*c*_ increases from 1 MPa·m^2^·s·mmol^−1^ (same order of magnitude as *R*) to 50 MPa·m^2^·s·mmol^−1^ (comparable to *R*_*a*_), which is a fifty times increase, the time constant τ is only increased by less than two times. These observations of a model leaf indicate that an effective strategy for a plant to be more resilient under water stress and to survive a drought would be to enlarge its water-storage capacitance, the ability to contain large amount of water, rather than to increase the resistance of pathways connecting xylem and capacitors. In a real-life plant, whose stomatal resistance is changeable and sensitive to the water status, a drought stressed condition and decreasing water content would trigger the closing of stomata, drastically increasing *R*_*a*_, which also prolongs the time constant and slows down the decrease of water potential (while ceasing transpiration), providing another effective strategy to overcome water stress.

### 3.3. Numerical Simulations of Leaf Water Status in Changing Environments

**Figure 5:**
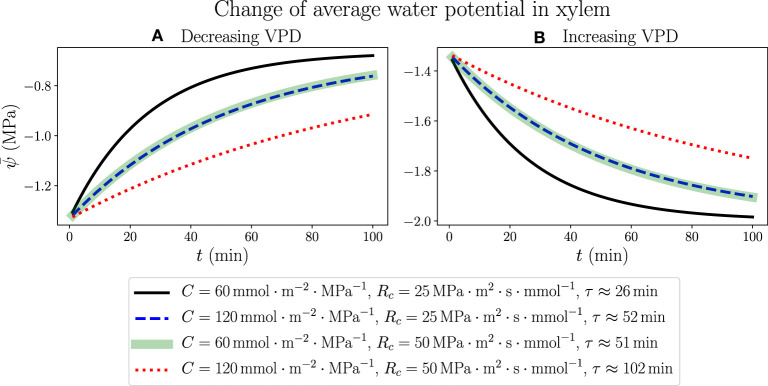
The time dependence of average water potential in xylem, when ψ_*a*_ is abruptly increased from −100MPa to −50MPa **(A)** where the air is wetted and VPD is decreased from 1.64 to 0.965kPa, or decreased from −100MPa to −150MPa **(B)** where the air is dried and VPD is increased from 1.64kPa to about 2.1kPa. Various sets of *C* and *R*_*c*_ values are used, while other parameters are the same as those in Figure 3. In both situations, the time constants τ corresponding to the same *C* and *R*_*c*_ values are identical as labeled in the legend.

We consider the response of a living, fully hydrated plant leaf to an instant change of atmospheric conditions, such as a sudden increase or decrease of relative humidity (RH) in the air, which is related to a rise or drop of atmospheric water potential through Equation (1). The results are generated by simulating the 100-node discretized network (with simulation time step Δ*t* = 0.01 *min*). We start from modeling a well-watered leaf in the steady state using the parameters for Figure 3 (ψ_*a*_ =−100 MPa, RH ≈ 48% and VPD ≈ 1.64 kPa), which is the same initial state in Figure 4, and at time *t* = 0 instantly change the value of ψ_*a*_, resulting in a continuous change of average water potential in xylem starting from −1.31 MPa as shown in Figure 5. As ψ_*a*_ is raised to −50 MPa, when the air is more humid (RH ≈ 70%) and the vapor pressure deficit across stomata becomes smaller (VPD ≈ 0.965 kPa), $\bar{\psi }$ also increases gradually toward a new steady-state value −0.656 MPa, which is determined by the new ψ_*a*_ value through Equations S7 and S8 in Supplementary Material with constant resistance values *R* and *R*_*a*_. Similarly, as ψ_*a*_ is dropped to −150 MPa, when the air is drier (RH ≈ 34%) and VPD becomes larger (2.1 kPa), $\bar{\psi }$ will decrease with time to an ultimate steady-state value −1.97 MPa. The analytical expressions for the time dependence of $\bar{\psi }$, as illustrated in Figures 5A,B for increasing and decreasing ψ_*a*_, respectively, are not explicitly available, but we can fit the profiles of $\bar{\psi }$ to exponential decay curves with time constant τ shown in the legend. The time constant for a certain set of *C* and *R*_*c*_ values turns out to be identical in both air wetting and drying situations, proving that the specific dynamics of leaf water status depends only on the internal hydraulic traits rather than external environments. In Supplementary Figure 3, the total transpiration rate *E* obtained in this modeling changes with time in a slightly different way, in which *E* would abruptly jump from its original steady-state value 1.97 mmol·m^−2^·s^−1^ to a new lower (decreasing VPD) or higher value (increasing VPD), and then gradually change with the same exponentially decaying trend and time constant as $\bar{\psi }$, to steady-state values 0.987 mmol·m^−2^·s^−1^ and 2.96 mmol·m^−2^·s^−1^ for cases in **(A)** and **(B)**, respectively.

The continuous change of average xylem water potential and the avoidance of drastic variation in a short time are another illustration of the functions of leaf water-storage capacitance *C*, as well as resistance *R*_*c*_ from xylem to capacitors, in stabilizing the plant hydraulics and reducing the variation of water content. The instant changes of ψ_*a*_ and VPD are used to model the effects of transient changes of wind condition, which would cause the leaf to lose water and dehydrate under the influence of a drying atmosphere even when the plant is well watered. It turns out that the effects of changing *C* and changing *R*_*c*_ on the time dependence of $\bar{\psi }$ are similar in this case within the ranges of parameters we choose. While making the capacitance twice as large will exactly increase the time constant to two times, suggesting a direct proportionality between *C* and τ, we find that doubling *R*_*c*_ will also make τ increase to a little lower than two folds. The weaker effect of *R*_*c*_ is possibly related to the presence of unchanging *R*_*a*_. This observation once again points to the effective strategy for a plant to overcome hydraulic destabilization and water loss due to negative environmental disturbances, by increasing either *C* or *R*_*c*_ of the leaf.

**Figure 6:**
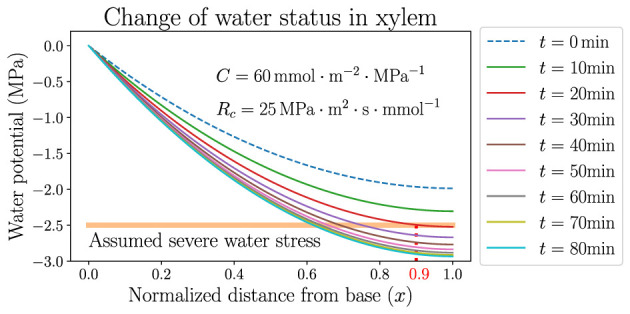
The temporal variation of spatially dependent water potential in xylem, when the atmospheric water potential ψ_*a*_ is instantly decreased from −100MPa to −150MPa where the air is dried. Parameters used are identical to those used for the black curves in Figure 5. The horizontal line labels the upper limit of presumed water potential under severe stress.

The stabilization of $\bar{\psi }$, however, only represents an average effect over the whole leaf from base to tip. The local xylem water potential ψ, which varies spatially in the leaf, would stay close to 0 near the base (*x* = 0) but would still become very negative near the tip (*x* = 1). An example is demonstrated in Figure 6, where the dynamics of spatially dependent water potential is simulated and its snapshots are plotted at multiple time points along the simulation. The figure shows the detailed changes of ψ according to the same hydraulic parameters and atmospheric condition of the black solid line in Figure 5B, where ψ_*a*_ decreases from –100 to −150 MPa instantly at *t* = 0 and VPD increases from 1.64 kPa to about 2.1 kPa. Within tens of minutes, the average potential $\bar{\psi }$ is stabilized at around −2 MPa (specifically −1.97 MPa) which is the new steady state at the lower ψ_*a*_, while the profile of ψ maintains a monotonically declining trend but lowers quantitatively with time. The rate of the lowering of ψ is initially faster and slows down later, corresponding to the exponential decay of $\bar{\psi }$. Even though the xylem average water potential is always higher than −2 MPa, the local water potential near the tip would experience severe water stress (which is assumed to be lower than −2.5 MPa here) after a short time. This severely stressed water potential is presumably lower than the value required for the normal functioning of a plant leaf, and would thoroughly dehydrate the leaf portion under this negative potential, making it lose physiological functions. For example, in about 20 min after the instant change of ψ_*a*_ and VPD, the leaf portion at *x* > 0.9 (between the vertical dotted line and the tip at *x* = 1.0 in Figure 6) would experience the low water potential and severe stress, and would be quickly dehydrated even when the average water status of the whole leaf is still relatively high. As the simulation proceeds with time, the leaf portion near the tip undergoing severe water stress would enlarge and the left boundary of this region (the vertical dotted line) would move to smaller *x*. This discovery calls for caution when studying the average water status of a leaf, which may be in a safe range for the leaf tissue to stay healthy, while the local water potential (especially at the tip) may be very negative and the leaf can be partly dehydrated, losing part of its functionality. A large capacitance *C*, and also large resistances *R*_*c*_ and *R*_*a*_, could help to delay the lowering of water potential by increasing the time scale τ, so that even the tip potential could be held at a high level to avoid dehydration for a relatively long time. A real-life living plant leaf would most likely close stomata and immediately raise *R*_*a*_, when facing dry wind in the air and going through quickly rising VPD.

### 3.4. Experimental Measurement Results of Water Content Distribution in *A. villosa* Leaves

**Figure 7:**
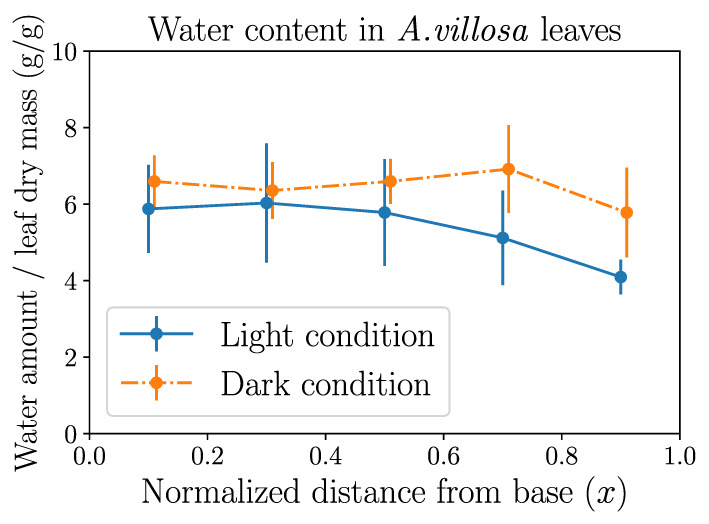
The experimental measurements of the mass of water content averaged over leaf dry mass (both in grams), in each of the five leaf portions of equal length. Six leaves of the grass *Anthaenantia villosa* in light conditions and six leaves of the same species in dark conditions are used in the measurements. Error bars represent standard deviations. A more even distribution in leaves in darkness and a tapering trend from base to tip in leaves exposed to light are observed. The two sets of data are plotted with a horizontal shift of 0.01 to show error bars.

The experimental data of the spatial variation of leaf water potential is usually difficult to obtain by using common techniques, while the measurement of local water content distribution is quantifiable by gravimetric methods. Figure 7 shows the different measurement results of local water amount averaged over leaf dry mass (to account for both leaf area and thickness) in leaves of the grass *A. villosa* when exposed to light or in dark conditions. It is observed that the leaves in darkness (where stomata are presumed to be closed) sustain a more evenly distributed water content, while leaves in light conditions (where stomata are presumed to be open) hold more water near the base but gradually decreasing water amount toward the tip, reflecting the declining trend of water potential. The statistical difference between the dark and light measurement results is most significant in the rightmost leaf segment nearest to the tip. Both observations as well as their difference actually demonstrate the usefulness of our modeling methods. The light treatment result indirectly confirms the finding in Figure 3. The dark treatment result, on the other hand, matches the prediction from Figure 1 that when stomata are closed and transpiration is stopped ($I_{i}^{\left(a\right)}=0$), in a fully hydrated steady state all currents (water flow) would also stop, leading to xylem water potential ψ_*i*_ = ψ_0_ throughout the xylem vessel and water content uniformly distributed along the leaf. See Supplementary Material for additional measurement data.

## 4. Discussion

One of the central assumptions of our theoretical modeling work on uniform grass leaf model is the constant resistance *R*_*a*_ from xylem to the atmosphere. This assumption implies that the stomatal resistance to water vapor flow is steady and independent of environmental changes within the modeled time period, a highly hypothetical stomatal behavior which is usually not accurate in a living plant. However, this idealized behavior is most helpful for focusing on the effects of water-storage capacitance and its associated resistance while avoiding the complication of a changeable stomatal resistance. This assumption is not trivial or baseless even from a plant biological point of view when studying short-term behaviors. It is shown that beginning with a well-watered state, both stomatal conductance and transpiration rate are stabilized and would not decrease significantly even when leaf water potential starts to drop, as long as the potential is higher than a threshold that causes stomata to react, increasing their resistance and ultimately closing (Brodribb, 2009; Choat et al., 2018). In fact, the dynamic processes modeled in this work all start from a well-watered steady state, and the results are meaningful for the study of initial changes and reactions of a leaf blade in response to instant or short-time variations of water conditions, which are exactly what is considered in the hypothesized dynamic scenarios.

In order to model the long-term dehydration dynamics, we would need to incorporate the dependence of stomatal resistance (and thus *R*_*a*_), on the leaf water status. The large *R*_*a*_ used in our modeling (compared with xylem hydraulic resistance) in the pathway from xylem to the atmosphere is mainly comprised of two parts, namely the stomatal resistance *R*_*s*_ and the outside-xylem resistance *R*_*ox*_ which is mostly through mesophyll and is also referred to as mesophyll resistance (Xiao and Zhu, 2017; Xiong et al., 2017). If we consider *R*_*a*_ as a variable dependent on water content *W*, we can assume *R*_*a*_(*W*) = *R*_*s*_(*W*) + *R*_*ox*_ in which *R*_*s*_ is a function of *W* and *R*_*ox*_ is a constant. We assume that the water content of the leaf when well-watered is *W*_0_, so that *R*_*s*_(*W*_0_) = 0 and *R*_*a*_(*W*_0_) = *R*_*ox*_ when the leaf is fully hydrated (*W* = *W*_0_) and stomata are open, and thus *R*_*ox*_ between xylem and stomata provides the minimum resistance in the transpiration pathway. The lumped model in Figure 2 is helpful for estimating the importance of the stomatal sensitivity to leaf water content (contained by capacitors) in controlling the transpiration rate. We suppose that when the leaf is slightly dehydrated from *W*_0_ to a water content *W* < *W*_0_, both whole-leaf lumped capacitor and stomatal resistance depend linearly on *W* (effectively expanded to the first order). For the linear capacitor, we have its voltage *V*(*W*) = *W*/*C* as a function of *W*, where *C* is the regular capacitance. For the linear stomatal resistance, we have *R*_*a*_(*W*) = *R*_*ox*_ + *s*(*W*_0_ − *W*) in which *R*_*s*_(*W*) = *s*(*W*_0_ − *W*) where *s* is a positive linear measure of the sensitivity of *R*_*a*_ (and *R*_*s*_) to *W*, so that *R*_*a*_ and *R*_*s*_ increase with decreasing *W* according to the expected behavior of real-life stomata. From Equations S22–S25 in Supplementary Material, we calculate the expressions of ψ_*x*_, *I*_*x*_, *I*_*a*_ and *I*_*c*_ (all found in Figure 2) in terms of given terminal water potentials (ψ_*p*_ and ψ_*a*_) and electrical traits including *V*(*W*) and *R*_*a*_(*W*). From the dependence of *I*_*c*_ on *W* and the relation *I*_*c*_ = d*W*/d*t*, we calculate a steady-state expression for *W* which represents the static water status of a living leaf. Furthermore, by substituting this steady-state *W* into the expression of *I*_*a*_, we obtain the following estimate of the relationship between terminal potentials and *I*_*a*_ (showing the highest-order term):

(18) $$\begin{array}{r} \psi_{p}-\psi_{a}\approx CR_{x}s\cdot I_{a}^{2}+\cdots \end{array}$$

which emphasizes the essential functions of leaf capacitance *C*, xylem hydraulic resistance *R*_*x*_ and the stomatal sensitivity to water content in limiting the increase of transpiration current *I*_*a*_ with an increasing water potential deficit ψ_*p*_ − ψ_*a*_ between inside and outside of the leaf. In this way both capacitance and stomatal control are shown to be helpful for keeping water content and reducing water loss in transpiration.

If the dehydration lasts longer and the leaf approaches severe stress (such as water potential below the limit of severe water tress in Figure 6), the xylem hydraulic conductance would also start to decrease due to the formation of embolism or cavitation, air bubbles blocking water flow in xylem vessels (Tyree and Ewers, 1991; Sack and Holbrook, 2006; Choat et al., 2012, 2018; Jones, 2013; Stroock et al., 2014). The dependence of xylem conductance on water potential is conventionally approximated as a logistic function with the shape of a sigmoid curve, in which the loss of conductance is negligible at relatively high potential and grows more rapidly with further lowering potential. Indeed, the incorporation of xylem and stomatal conductances dependent on water potential is applied in several theoretical models, in which the stomatal dependence is also treated as sigmoidal or approximately piecewise linear functions (Mencuccini et al., 2019). Alternatively, a more direct stomatal dependence on VPD can also be established and implemented (Grossiord et al., 2020). Most recently, the sigmoidal behaviors of both xylem and stomatal conductances are applied in a spatially explicit study (Jain et al., 2021). The specific biophysical and biochemical mechanisms that control stomatal opening through water status, including the turgor pressure of guard cells and epidermal cells around stomata and the use of plant hormone abscisic acid, are broadly explored by existing literature (Buckley, 2005). If the spatial variation of stomatal conductance (or resistance) along leaf surface is also included, the model would need to take into account anatomical data over leaf blade, including sizes and spatial distributions of stomata and xylem vessels (Ocheltree et al., 2012; Rockwell and Holbrook, 2017). It would be necessary to incorporate not only the overall dependences of whole-system hydraulic traits on average water status in the large scale (such as sigmoid), but also small-scale quantitative relationships between stomatal and xylem resistances at fine spatial resolution and their local water potential or content, to make full use of our spatially explicit capacitive model. We expect to experimentally measure these fine-resolution quantities and implement the experimental inputs in future modeling. For the completeness of the current work, we have uploaded the source code of grass leaf simulations for interested readers to try simulating leaves with extra spatial variations or stomatal features. (See the data availability statement.)

Under the assumption of constant stomatal opening, our computation and simulation results indicate that both capacitance *C* and the resistance *R*_*c*_ from xylem to capacitors play significant roles in defining the time constant τ, which determines the rate of change in a dehydration (or hydration) dynamics. Both *C* and *R*_*c*_ are positively related to τ, and thus a plant with large *C* or *R*_*c*_ can effectively slow down dehydration under short-term water stresses and maintain leaf-water potential higher than the threshold which causes stomata to close and so that photosynthesis and other physiological functioning can proceed. Similar time constants (as product of whole-system hydraulic resistance and capacitance) were described in previous literature (Chuang et al., 2006; Meinzer et al., 2009). This maintenance of water status can be observed in experimental studies of grass leaves. Specifically, in a leaf that dehydrates slowly (possibly due to larger capacitance), stomatal conductance would change less with atmospheric humidity and VPD by a smaller magnitude of slope compared to a leaf showing faster dehydration dynamics. The two types of water-storage cells in grass leaves, bulliform and bundle sheath, are different in their specific capacitance values *C*, which are related to cell wall rigidity or elasticity, and also in *R*_*c*_ values. Bundle sheaths are much closer to xylem conduits which are contained in vascular bundles, while bulliform cells are mostly distributed in the epidermis on the upper side of a leaf. The longer water pathways from xylem to bulliform may lead to larger *R*_*c*_ and greater contribution to the delay of water loss. In our simulation, using two sets of capacitors and resistors could be feasible when dealing with the cell types. In certain plants, a variable capacitance depending on water status was observed (Salomón et al., 2017) and could be additionally applied. The baseline osmotic potential ψ_*s*_ of water storage, which is determined by the most negative osmotic potential due to the presence of aqueous solutes in leaf cells and does not play an explicit part in this study, can be obtained by measuring *C* and water content *W* = *C*(ψ_*x*_ − ψ_*s*_) where ψ_*x*_ is the steady-state mean potential in xylem. These measurements can be achieved through pressure-volume (PV) curves, which measure the interdependence between leaf potential and water content (Abrams, 1988; Jones, 2013). Another factor worth considering when studying water flows outside of xylem, both to stomata for transpiration or to leaf cells for storage, is the actual form of water transport. Recent modeling efforts have specifically investigated the relative importance of symplastic (through cell cytoplasm), apoplastic (inside cell wall but outside cell membrane), and gaseous pathways (especially in transpiration), as well as the exact site of exiting water evaporation (either near vascular bundle or near stomata and epidermis) which may complicate the hydraulic condition inside a leaf (Rockwell et al., 2014; Buckley, 2015; Buckley et al., 2017).

Our spatially explicit modeling methods provide a new theoretical approach to the study of fluid dynamics of general flow networks with fluid-storage function. Such networks are not necessarily hydraulic vascular networks found in plant leaves, but could also be found in other water transport systems such as river networks. The applicability of the modeling methods to plant biology has been illustrated in the computation and simulation results of grass leaf hydraulics. The use of grass leaves as a model benefits from not only simpler stomata and venation arrangements than dicots, but also the clear presence of water-storage cells (capacitors). The spatially dependent xylem water potential profile, which decreases from base to tip in well-watered steady state (Figure 3) when capacitors are static, compares qualitatively well with earlier calculation results of wheat leaves by Altus et al. (1985) and most recent experimental measurements (using a novel method) and theoretical predictions of maize leaves by Jain et al. (2021), as well as water potential gradients along sugarcane stems (Meinzer et al., 1992). By tuning the biologically relevant parameters we choose, we can reproduce quantitatively matching results, though these previous studies considered local resistances without explicit water storage. The measurement results of the spatial distribution of water content in *A. villosa* leaves in Figure 7, which are statistically different toward leaf tip between light and dark conditions, also support key predictions and the effectiveness of the model.

To further improve the ability of our model to accurately predict leaf hydraulic behaviors, we can implement the grass leaf vascular architecture with hierarchy, where major lateral veins and minor intermediate veins are parallel and connected by transverse veins, as shown by Altus et al. In fact, hierarchical structures exist among all plant leaf networks, especially dicotyledonous leaves whose veins are not parallel but instead form branches and loops. It has been experimentally found in dicot leaves that major veins (with high conductivity) are useful for distributing water throughout the leaf blade evenly in a fast manner, and minor veins (with high resistivity) are used to deliver water to leaf cells (Zwieniecki et al., 2002). This observation has also been simulated by a resistor-only model to verify the functions of multiple levels of veins with different resistances in a mesh network (Cochard et al., 2004). Our modeling could help to reveal the function of capacitance in water flow dynamics and balancing of water distribution in similar networked systems. At each level of the hierarchy, the continuous anatomical narrowing of xylem conduits from leaf base to tip can also affect the water potential pattern (Lechthaler et al., 2020) and can be implemented in our model with a large number of nodes. Along with hierarchical considerations, and guided by empirical sub-leaf scale data, we plan to apply more biologically realistic anatomical and physiological inputs in future simulation studies based on our current model. We can then gain a better understanding of the importance of capacitance in time-dependent leaf hydraulic behavior in both natural and agricultural settings to help explain evolutionary adaptations of plants to manage water, and, eventually inspire more efficient agricultural practices.

## Data Availability Statement

The original contributions presented in the study are publicly available. The code used for the numerical simulations can be found at https://github.com/yongtianluo/Leaf-capacitive-hydraulics.

## Author Contributions

YL developed the theoretical methods, analyzed the results, and wrote the manuscript. C-LH performed the experiments. BRH designed the research and revised the manuscript. EK conceived and designed the research and revised the manuscript. All authors contributed to the article and approved the submitted version.

## Funding

The authors acknowledge support from NSF-IOS, Award 1856587.

## Conflict of Interest

The authors declare that the research was conducted in the absence of any commercial or financial relationships that could be construed as a potential conflict of interest.

## Publisher's Note

All claims expressed in this article are solely those of the authors and do not necessarily represent those of their affiliated organizations, or those of the publisher, the editors and the reviewers. Any product that may be evaluated in this article, or claim that may be made by its manufacturer, is not guaranteed or endorsed by the publisher.
