# Supplementary material for: Leaf Water Storage and Robustness to Intermittent Drought: A Spatially Explicit Capacitive Model for Leaf Hydraulics
Source: Front Plant Sci. 2021 Oct 14;12:725995. doi: 10.3389/fpls.2021.725995 (PMC8551678; doi:10.3389/fpls.2021.725995)
Supplement: Supplementary file 1 [file Data_Sheet_1.pdf]

Supplementary Material

1 GENERAL ANALYTICAL SOLUTION OF THE 1D CONTINUOUS MODEL

In order to solve Equation (8), we separate the xylem water potential into a static part and a time-dependent part, $\psi(x,t) = \psi_u(x) + \psi_t(x,t)$, where the two parts satisfy:

$$\frac{\partial^2 \psi_u}{\partial x^2} = \frac{R}{R_a}\psi_u - \frac{R\psi_a}{R_a} \tag{S1}$$

$$\frac{\partial^3 \psi_t}{\partial t \partial x^2} = R(\frac{1}{R_a} + \frac{1}{R_c})\frac{\partial \psi_t}{\partial t} - \frac{1}{CR_c}\frac{\partial^2 \psi_t}{\partial x^2} + \frac{R}{CR_cR_a}\psi_t. \tag{S2}$$

Given the boundary conditions $\psi_u(x=0)$ which is a constant and $I(x=1)=0$, the time-independent ψ_u is solved as:

$$\psi_u(x) = \frac{\psi_u(0) - \psi_a}{1 + \exp(2\sqrt{R/R_a})}\exp\left(\sqrt{\frac{R}{R_a}}x\right) + \frac{\psi_u(0) - \psi_a}{1 + \exp(-2\sqrt{R/R_a})}\exp\left(-\sqrt{\frac{R}{R_a}}x\right) + \psi_a. \tag{S3}$$

The time-dependent ψ_t can be solved through a Fourier transform of the water potential $\tilde{\psi}_t(x,\omega) = 1/\sqrt{2\pi}\int \mathrm{d}t\, e^{-i\omega t}\psi_t(x,t)$ (Asmar, 2005) which satisfies:

$$i\omega\frac{\partial^2 \tilde{\psi}_t}{\partial x^2} = i\omega R\left(\frac{1}{R_a} + \frac{1}{R_c}\right)\tilde{\psi}_t - \frac{1}{CR_c}\frac{\partial^2 \tilde{\psi}_t}{\partial x^2} + \frac{R}{CR_cR_a}\tilde{\psi}_t \tag{S4}$$

whose solution is

$$\tilde{\psi}_t(x,\omega) = \alpha(\omega)\exp(\kappa(\omega)x) + \beta(\omega)\exp(-\kappa(\omega)x) \tag{S5}$$

$$\kappa(\omega) = \sqrt{\frac{i\omega CR(R_c/R_a + 1) + R/R_a}{1 + i\omega CR_c}} \tag{S6}$$

where the functions $\alpha(\omega)$ and $\beta(\omega)$ can be determined by given boundary conditions $\psi_t(x=0,t)$ and $I(x=1)=0$. If $\psi(x=0)=\psi_0$ is time-independent, $\psi_t(x=0,t)=0$ and the water status will stay in the steady state ψ_u described by Equation (S3). The steady-state average potential in the xylem is:

$$\bar{\psi} = \int_0^1 \mathrm{d}x\,\psi_u(x) = I_0R_a + \psi_a \tag{S7}$$

where $I_0 = I(x=0)$ is the current entering through the base:

$$I_0 = -\frac{1}{R}\frac{\partial \psi_u}{\partial x}\bigg|_{x=0} = \frac{\exp(2\sqrt{R/R_a}) - 1}{\exp(2\sqrt{R/R_a}) + 1}\cdot\frac{\psi_u(0) - \psi_a}{\sqrt{RR_a}} \tag{S8}$$

which is equal to the total transpiration current E at steady state.

2 ANALYTICAL SOLUTION OF THE 1D CONTINUOUS MODEL WITH AN OSCILLATING WATER POTENTIAL AT BASE

In a uniform, continuous, one-dimensional model described by Equation (8), we solved its steady-state water potential distribution $\psi_u(x)$ in Equation (S3). Here we present the solution of an oscillating boundary condition $\psi_t(x=0,t) = A\cos(\omega_0 t + \varphi)$, whose Fourier transform is:

$$\tilde{\psi}_t(x=0,\omega) = A\sqrt{\frac{\pi}{2}}[e^{i\varphi}\delta(\omega-\omega_0) + e^{-i\varphi}\delta(\omega+\omega_0)] \tag{S9}$$

where δ is the Dirac delta. From the form of solution $\tilde{\psi}_t(x,\omega) = \alpha(\omega)\exp(\kappa(\omega)x) + \beta(\omega)\exp(-\kappa(\omega)x)$ in Equation (S5), we have the following boundary conditions: $\alpha(\omega) + \beta(\omega) = A\sqrt{\pi/2}[e^{i\varphi}\delta(\omega-\omega_0) + e^{-i\varphi}\delta(\omega+\omega_0)]$ and $\beta(\omega)\exp(-\kappa(\omega)) - \alpha(\omega)\exp(\kappa(\omega)) = 0$, the latter of which is derived from the fact that $I = -(1/R)\partial\psi/\partial x$ and $I(x=1) = 0$. The functions α and β are solved to be:

$$\alpha(\omega) = A\sqrt{\frac{\pi}{2}}\frac{e^{i\varphi}\delta(\omega-\omega_0) + e^{-i\varphi}\delta(\omega+\omega_0)}{1+\exp(2\kappa(\omega))} \tag{S10}$$

$$\beta(\omega) = A\sqrt{\frac{\pi}{2}}\frac{e^{i\varphi}\delta(\omega-\omega_0) + e^{-i\varphi}\delta(\omega+\omega_0)}{1+\exp(-2\kappa(\omega))}. \tag{S11}$$

We substitute the expressions into $\tilde{\psi}_t(x,\omega)$, which can be inversely transformed into the real time ($\psi_t = 1/\sqrt{2\pi}\int \mathrm{d}\omega\, e^{i\omega t}\tilde{\psi}_t$):

$$\begin{aligned}\psi_t(x,t) =& \frac{A}{2}\Big\{e^{i(\omega_0 t+\varphi)}\left[\frac{\exp(\kappa(\omega_0)x)}{1+\exp(2\kappa(\omega_0))} + \frac{\exp(-\kappa(\omega_0)x)}{1+\exp(-2\kappa(\omega_0))}\right] \\ &+ e^{-i(\omega_0 t+\varphi)}\left[\frac{\exp(\kappa(-\omega_0)x)}{1+\exp(2\kappa(-\omega_0))} + \frac{\exp(-\kappa(-\omega_0)x)}{1+\exp(-2\kappa(-\omega_0))}\right]\Big\}\end{aligned} \tag{S12}$$

where function $\kappa(\omega) = \sqrt{[i\omega CR(R_c/R_a+1) + R/R_a]/(1+i\omega CR_c)}$ as in Equation (S6). The total water potential distribution is then $\psi(x,t) = \psi_u(x) + \psi_t(x,t)$ (Equations (S3)+(S12)).

3 ANALYTICAL CALCULATION OF A UNIFORM XYLEM NETWORK REMOVED FROM PLANT

We consider a uniform, continuous xylem model which is at the steady state (Equation (S3)) when $t < 0$, and is removed from base water source when $t = 0$. A closed end immediately forms at $x = 0$ which is similar to the terminal at $x = 1$. In Figure 1 at the first node $i = 1$ of the network, $I_{0,1}$ becomes zero instantly and we have $I_{1,2} + I_1^{(a)} + I_1^{(c)} = 0$, which means $\partial I_{1,2}/\partial t = -\partial I_1^{(a)}/\partial t - \partial I_1^{(c)}/\partial t$ and that:

$$\frac{\partial I_{1,2}}{\partial t} = -\Big(\frac{1}{R_a} + \frac{1}{R_c}\Big)\frac{\partial\psi_1}{\partial t}\Delta x - \frac{1}{CR_c}\Big(I_{1,2} + \frac{\psi_1 - \psi_a}{R_a}\Delta x\Big). \tag{S13}$$

At the continuous limit, $\Delta x \to 0$ and $I_{1,2} \to I(x=0)$, and the equation becomes $\partial I(0)/\partial t = -I(0)/(CR_c)$. Along with the initial condition $I(x=0, t<0) = I_0$ in Equation (S8), we obtain:

$$I(x=0,t) = I_0 - I_0\Big(1 - \exp(-\frac{t}{CR_c})\Big)H(t) \tag{S14}$$

where H is the Heaviside step function. This equation shows that the current at $x = 0$ does not become zero instantly because of the existence of capacitance. The time-dependent part of $I(x = 0, t)$ is $I_t(x = 0, t) = -I_0[1 - \exp(-t/(CR_c))]H(t)$, whose Fourier transform is:

$$\tilde{I}_t(x = 0, \omega) = -I_0\sqrt{\frac{\pi}{2}}\Big(\delta(\omega) + \frac{1}{i\pi\omega(i\omega CR_c + 1)}\Big). \tag{S15}$$

From $I = -(1/R)\partial\psi/\partial x$ and $I(x = 1) = 0$, we can calculate the Fourier transform of the time-dependent part of xylem water potential $\tilde{\psi}_t(x, \omega) = \alpha(\omega)\exp(\kappa(\omega)x) + \beta(\omega)\exp(-\kappa(\omega)x)$ in which:

$$\alpha(\omega) = -\sqrt{\frac{\pi}{2}}\frac{I_0 R}{\kappa(\omega)[\exp(2\kappa(\omega)) - 1]}\Big(\delta(\omega) + \frac{1}{i\pi\omega(i\omega CR_c + 1)}\Big) \tag{S16}$$

$$\beta(\omega) = -\sqrt{\frac{\pi}{2}}\frac{I_0 R}{\kappa(\omega)[1 - \exp(-2\kappa(\omega))]}\Big(\delta(\omega) + \frac{1}{i\pi\omega(i\omega CR_c + 1)}\Big). \tag{S17}$$

The xylem water potential distribution in the real time is not analytically solvable. However, we can instead calculate the time-dependent part of the average xylem potential, whose Fourier transform is:

$$\tilde{\psi}_{\text{avg}}(x, \omega) = \int_0^1 \mathrm{d}x\, \tilde{\psi}_t(x, \omega) = -\sqrt{\frac{\pi}{2}}\frac{I_0(1 + i\omega CR_c)}{i\omega C(R_c/R_a + 1) + 1/R_a}\left(\delta(\omega) + \frac{1}{i\pi\omega(i\omega CR_c + 1)}\right). \tag{S18}$$

The inverse Fourier transform gives

$$\frac{1}{\sqrt{2\pi}}\int \mathrm{d}\omega\, e^{i\omega t}\tilde{\psi}_{\text{avg}}(x, \omega) = -\frac{I_0 R_a}{2} - I_0 R_a\Big[1 - \exp\Big(-\frac{t}{C(R_c + R_a)}\Big)\Big]H(t). \tag{S19}$$

Because of the continuity of $\bar{\psi}$ at $t = 0$, which is equal to its steady-state value in Equation (S7), the average xylem potential in real time is:

$$\bar{\psi} = \psi_a + I_0 R_a - I_0 R_a\Big[1 - \exp\Big(-\frac{t}{C(R_c + R_a)}\Big)\Big]H(t) \tag{S20}$$

and the total transpiration current is calculated as:

$$E = \int_0^1 \mathrm{d}x\, \frac{\psi(x, t) - \psi_a}{R_a} = \frac{\bar{\psi} - \psi_a}{R_a} = I_0 - I_0\Big[1 - \exp\Big(-\frac{t}{C(R_c + R_a)}\Big)\Big]H(t). \tag{S21}$$

Both equations result in Expressions (10) and (11) when $t > 0$.

4 THE LUMPED MODEL AND ITS PARAMETER SELECTION

We compare the basic functions of the one-dimensional model in Figure 1 (left) with those of the lumped model in Figure 2 (right):

$$I_{0,1} = \sum_i I_i^{(a)} + \sum_i I_i^{(c)} \qquad\qquad I_x = I_a + I_c \tag{S22}$$

$$\psi_0 - \psi_N = \sum_i R_{i-1,i} I_{i-1,i} \qquad\qquad \psi_p - \psi_x = R_x I_x \tag{S23}$$

$$\bar{\psi} - \psi_a = \frac{\sum_i R_i^{(a)} I_i^{(a)}}{N} \qquad\qquad \psi_x - \psi_a = R_a I_a \tag{S24}$$

$$\bar{\psi} - \bar{V} - \psi_s = \frac{\sum_i R_i^{(c)} I_i^{(c)}}{N} \qquad\qquad \psi_x - V - \psi_s = R_c I_c \tag{S25}$$

where $\bar{\psi} = \sum_i \psi_i/N$ and $\bar{V} = \sum_i V_i/N$. We investigate the equivalence of the two sets of equations by defining $I_x = I_{0,1}$, $I_a = \sum_i I_i^{(a)}$, $I_c = \sum_i I_i^{(c)}$, $\psi_x = \bar{\psi}$ and $V = \bar{V}$. In Subsection 2.2, for a uniform one-dimensional network where circuit elements are evenly distributed, we have defined R_a and R_c as grouped elements for the whole system, and these definitions will make the equations in (S22), (S24) and (S25) equivalent. To make the equations in (S23) also equivalent, we study how R_x is related to $R = \sum_i R_{i-1,i}$. We already defined $\psi_p = \psi_0$, and here we use the steady state of a uniform 1D model that was calculated in Subsection 2.2 to compare the left hand sides of the equations, which are $\psi_u(x=0) - \psi_u(x=1)$ and $\psi_u(x=0) - \bar{\psi}$ in the 1D model. We estimate their comparison by doing:

$$\frac{\psi_u(x=0) - \psi_u(x=1)}{\psi_u(x=0) - \bar{\psi}} = \frac{1 + \exp(2\sqrt{R/R_a}) - 2\exp(\sqrt{R/R_a})}{1 + \exp(2\sqrt{R/R_a}) - \sqrt{R_a/R}(\exp(2\sqrt{R/R_a}) - 1)} \approx \frac{3}{2}. \tag{S26}$$

The typical outside-xylem resistance is larger than the resistance in the xylem, i.e. $R_a \gtrsim R$, and we obtain the approximate ratio $3/2$. To compare the right hand sides, which in the uniform model are $R\bar{I}$ where $\bar{I} = (\psi(x=0) - \psi(x=1))/R$ and $R_x I_x$ where $I_x = I_0$, we estimate the ratio:

$$\frac{\bar{I}}{I_x} = \sqrt{\frac{R_a}{R}} \frac{\exp(\sqrt{R/R_a}) + \exp(-\sqrt{R/R_a}) - 2}{\exp(\sqrt{R/R_a}) - \exp(-\sqrt{R/R_a})} \approx \frac{1}{2} \tag{S27}$$

provided that $R_a \gtrsim R$. Combining the two pairs of comparisons, we found that for equations in (S23) to be compatible with each other, we could have $R_x = R/3$ which is $1/3$ the total xylem resistance from base to tip.

The lumped model in Figure 2 is described by the dynamic equation:

$$\left(\frac{1}{R_a} + \frac{1}{R_x} + \frac{1}{R_c}\right)\frac{\partial \psi_x}{\partial t} = -\frac{1}{CR_c}\left(\frac{1}{R_a} + \frac{1}{R_x}\right)\psi_x + \frac{1}{CR_c}\left(\frac{\psi_a}{R_a} + \frac{\psi_p}{R_x}\right) \tag{S28}$$

whose solution for ψ_x is:

$$\psi_x = \frac{\frac{\psi_a}{R_a} + \frac{\psi_p}{R_x}}{\frac{1}{R_a} + \frac{1}{R_x}} + D\exp\left(-\frac{\frac{1}{CR_c}\left(\frac{1}{R_a} + \frac{1}{R_x}\right)}{\frac{1}{R_a} + \frac{1}{R_x} + \frac{1}{R_c}} t\right) \tag{S29}$$

where the first part is the steady state and the second part is the dynamics, where the parameter D is the difference between the initial state and the steady state. The time constant becomes $\tau = C(R_c + R_a)$ if the model describes a leaf removed from plant ($R_x \to \infty$), the same result as in the uniform network model (Subsection 2.2).

5 SUPPLEMENTARY THEORETICAL MODELING RESULTS OF UNIFORM GRASS LEAVES WITH CONSTANT PARAMETERS

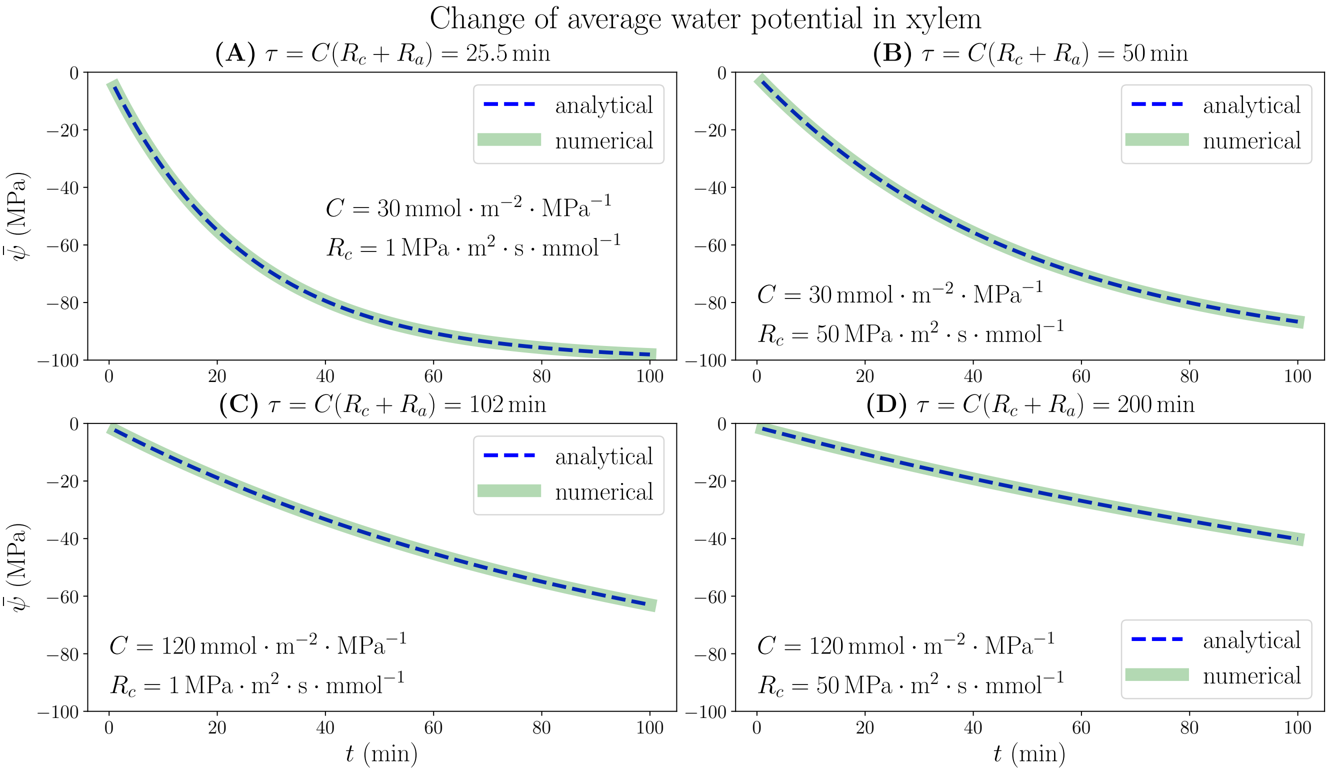

Figure S1: The time dependence of average xylem potential when a living, uniform model leaf in steady state is removed from plant and water source is closed at the base. The 1D simulation results match the analytical exponential decay expressions, with time constants $\tau = C(R_c + R_a)$. The assumption of constant stomatal resistance (and constant R_a), which leads to very negative $\bar{\psi}$ in the long term, is highly hypothetical, but is helpful for theoretical investigation. The temporal variations of total transpiration rate are similar. Various sets of whole-leaf capacitance C and xylem-to-capacitor resistance R_c values are used, while other parameters are the same as those in Figure 3 in the main text.

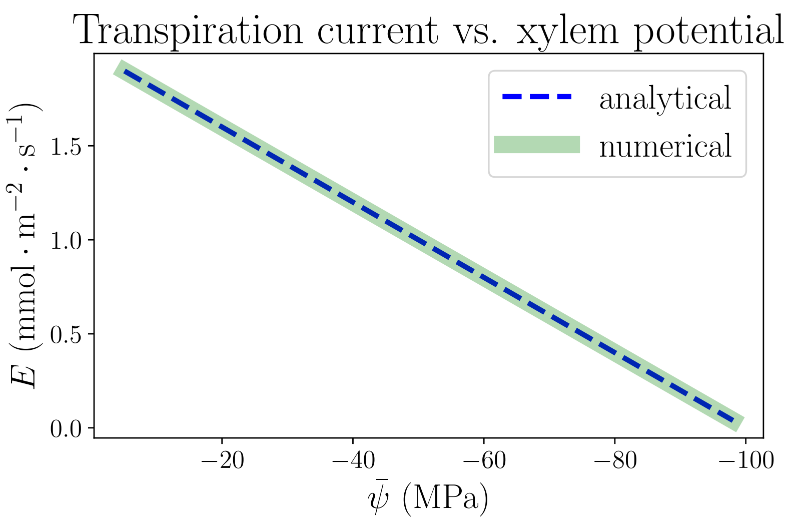

Figure S2: The dependence of total transpiration rate E on average xylem water potential $\bar{\psi}$ in the modeling of an excised leaf. The decrease of E is induced by the lowering of $\bar{\psi}$ because of open stomata and constant transpiration resistance R_a. Parameters are the same as those in Figure 3 in the main text.

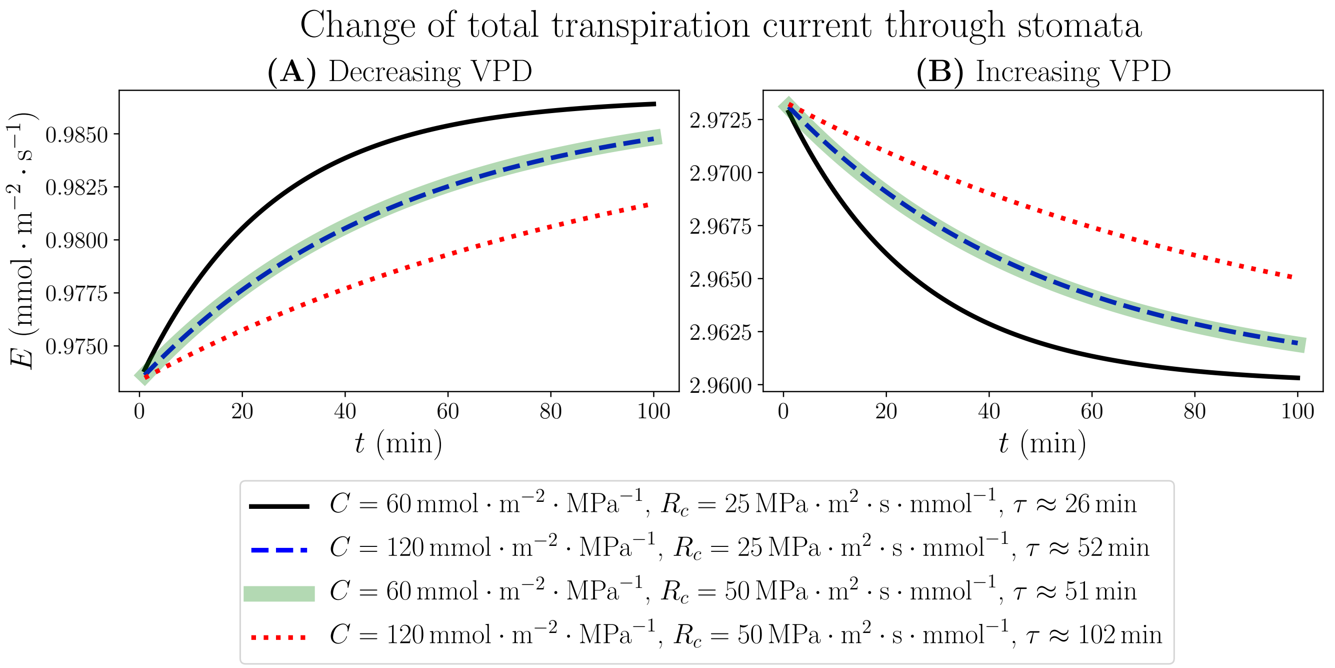

Figure S3: The time dependence of total transpiration rate through stomata, when ψ_a is instantly increased from $-100\,\mathrm{MPa}$ to $-50\,\mathrm{MPa}$ **(A)** where the air is wetted and VPD is decreased from $1.64\,\mathrm{kPa}$ to $0.965\,\mathrm{kPa}$, or decreased from $-100\,\mathrm{MPa}$ to $-150\,\mathrm{MPa}$ **(B)** where the air is dried and VPD is increased from $1.64\,\mathrm{kPa}$ to about $2.1\,\mathrm{kPa}$. Various sets of C and R_c values are used, while other parameters are the same as those in Figure 3 in the main text. In both situations, the time constants τ corresponding to the same C and R_c values are identical as labeled in the legend. Note that in both **(A)** and **(B)** the original transpiration rate at $t < 0$ is $E = 1.97\,\mathrm{mmol} \cdot \mathrm{m}^{-2} \cdot \mathrm{s}^{-1}$, which undergoes an instant change at $t = 0$ and then slightly changes in an exponential way when $t > 0$.

6 SUPPLEMENTARY MEASUREMENTS OF THE SPATIAL DISTRIBUTION OF WATER CONTENT ALONG *A. VILLOSA* LEAF BLADES

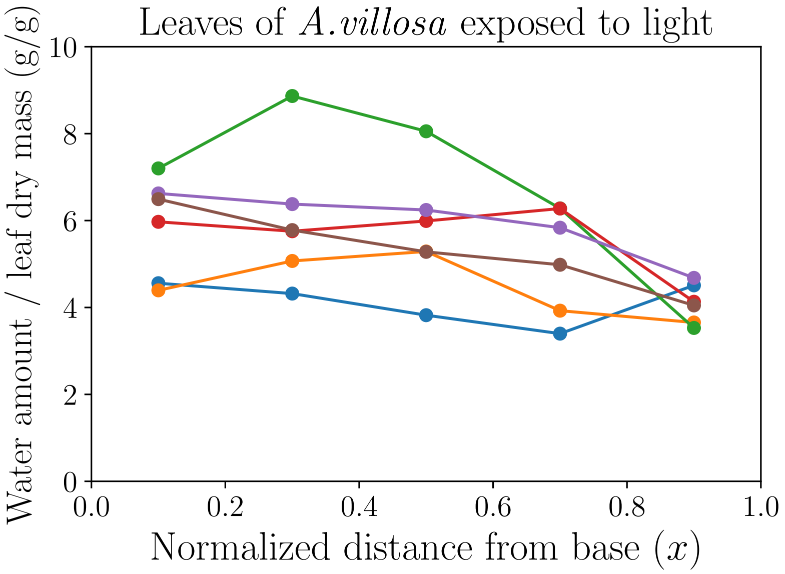

Figure S4: Raw data of local water amount measurements for six *A. villosa* leaves under light treatment.

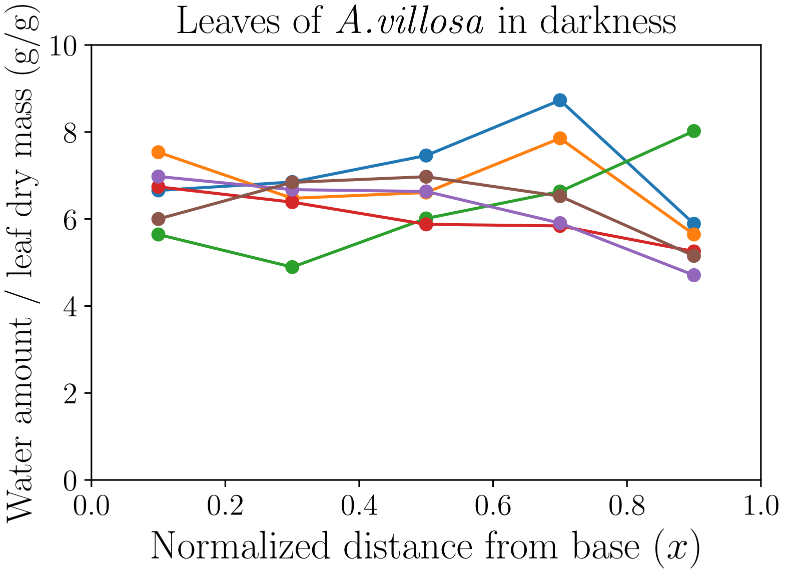

Figure S5: Raw data of local water amount measurements for six *A. villosa* leaves under dark treatment.

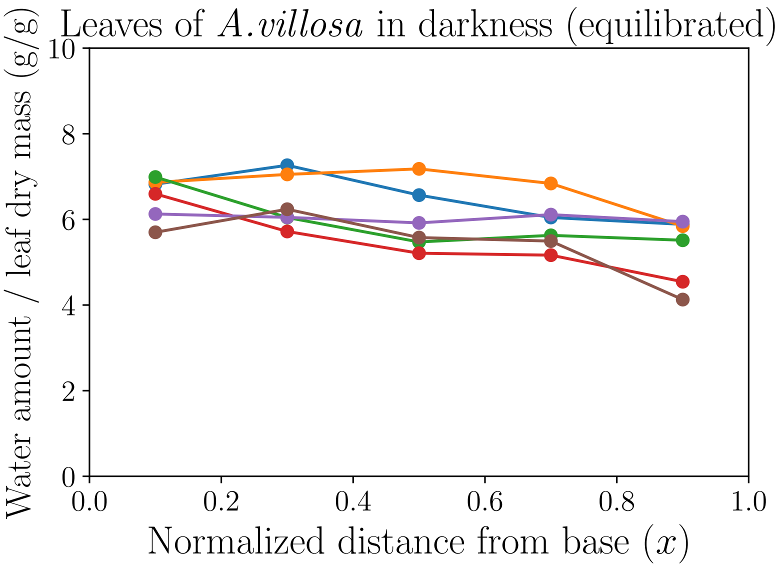

Figure S6: Raw data of local water amount measurements for six *A. villosa* leaves under dark + 1 hour equilibrium treatment.

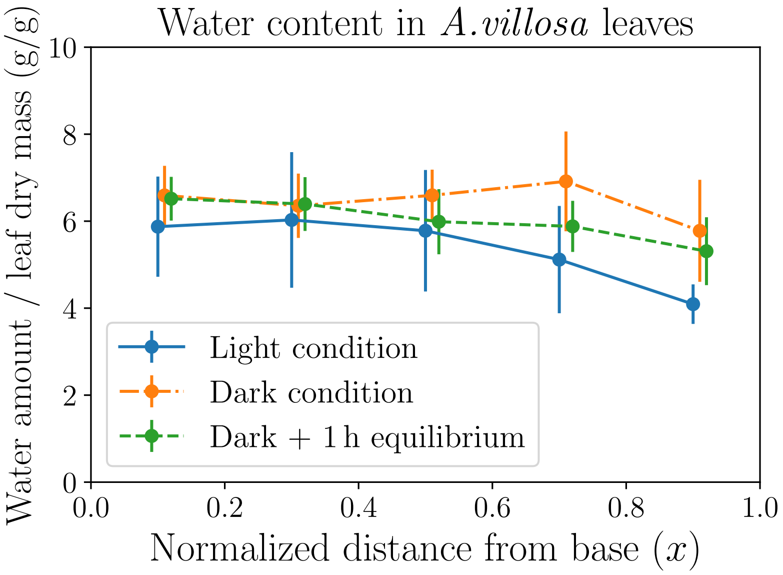

Figure S7: The combined measurement results of *A. villosa* leaves under three different treatments. Error bars represent standard deviations. A more even distribution in leaves in darkness and a tapering trend from base to tip in leaves exposed to light are observed. The leaves equilibrated for an extra hour after excision do not show statistically significant difference from dark treatment results. The three data sets are plotted with a horizontal shift of 0.01 to show error bars.

REFERENCES

Asmar, N. H. (2005). *Partial Differential Equations with Fourier Series and Boundary Value Problems* (Pearson Prentice Hall), 2nd edn.
